# Correlation analysis of Type-2 diabetes mellitus with proliferative retinopathy and central macular thickness

**DOI:** 10.12669/pjms.40.4.7726

**Published:** 2024

**Authors:** He Huang, Shuang Jiang, Honglei Niu

**Affiliations:** 1 He Huang, Department of Ophthalmology, Third Affiliated Hospital of Jinzhou Medical University, Jinzhou, Liaoning, 121000, China. Department of Fundus, Aier Eye Hospital (Taiyuan), Tiyuan 030000, Shanxi, China; 2 Shuang Jiang, Department of Ophthalmology, Third Affiliated Hospital of Jinzhou Medical University, Jinzhou, Liaoning, 121000, China; 3 Honglei Niu, Department of Vitreoretinae, Shanxi Eye Hospital, Tiyuan 030000, Shanxi, China

**Keywords:** Type-2 diabetes mellitus, Proliferative diabetic retinopathy, Central macular thickness, Risk factor, Logistic regression, ROC curve

## Abstract

**Objective:**

To investigate the relevant risk factors of proliferative diabetic retinopathy (PDR) in patients with Type-2 diabetes mellitus (T2DM) and their correlations with the central macular thickness (CMT).

**Methods:**

This is a retrospective study. The clinical data of 300 patients with T2DM were collected and divided into a PDR group (observation group) and non-PDR group (control group) according to the occurrence of PDR in Aier Eye Hospital (Taiyuan) from February 2019 to February 2022. The relevant risk factors were screened out through the t test and the χ^2^ test, and analyzed by logistic regression.

**Results:**

Logistic regression analysis showed that systolic blood pressure, diastolic blood pressure, course of diabetes, fasting blood glucose (FBG), two hours postprandial blood glucose (two hours PBG) and urinary albumin were independent risk factors for T2DM complicated with PDR. ROC curve revealed that systolic blood pressure, course of diabetes and urinary albumin had the highest diagnostic efficiency. Correlation analysis demonstrated that CMT was positively correlated with systolic blood pressure, course of diabetes, HbA1c level and urinary albumin level.

**Conclusion:**

For patients with T2DM, blood pressure, course of diabetes, FBG, 2hPBG and urinary albumin are independent risk factors for PDR, and increased systolic blood pressure, course of diabetes, HbA1c level and urinary albumin level will increase CMT. Combining the above indexes to predict the occurrence of PDR has a synergistic effect, and the increase in systolic blood pressure, course of diabetes, HbA1c level and urinary albumin level will increase the CMT of the patients.

## INTRODUCTION

Diabetes is a progressive multisystem endocrine disease that seriously endangers human health. Its complications are numerous and its prognosis is poor, with mortality and disability only second to cancers and cardio-cerebrovascular diseases. It is estimated that by 2030, the proportion of diabetic patients in the world will reach 7.7%, and the number will increase to 552 million.^1^,^2^ Diabetic retinopathy (DR) is one of the most common chronic microvascular complications of diabetes. With the increase in the incidence of diabetes worldwide, the global prevalence of DR has also climbed to 34.6%.^3^ According to statistics, the incidence of DR in diabetic patients in China is also as high as 22.4%.^4^

Diabetic patients are prone to visual impairment and even blindness after DR, which is the main cause of blindness and seriously affects the quality of life in diabetic patients. After diabetic patients suffers from complications like DR, with the progression of the disease and the continuous existence of microvascular dysfunction, the density of blood vessels decreases abnormally, resulting in insufficient substance supply needed for retinal metabolism. Consequently, DR can progress to proliferative diabetic retinopathy (PDR), which is the main stage leading to a sharp decline in patients’ vision, characterized by progressive destruction of microvascular structure with long-term exposure to a hyperglycemic microenvironment.^5^

The destruction of microvascular structure will damage the blood-retinal barrier, resulting in hypoxia and microcirculation disturbance of the retina. Moreover, with the progression of PDR, the patients’ retina is prone to hemorrhage, accompanied by the formation of preretinal fibrovascular membrane, which will lead to retinal traction, thereby causing vitreous hemorrhage and tractional retinal detachment, and resulting in a significant decline in the patients’ vision and even blindness. Diabetic macular edema (DME) is the main cause of visual impairment. Persistent hyperglycemia can cause retinal hypoxia, produce a variety of inflammatory factors, destroy the blood-retinal barrier and change vascular permeability, leading to changes in central macular thickness (CMT), and ultimately blindness.

The pathogenesis of PDR is affected by multiple factors, such as polyol metabolic pathway, protein kinase pathway and advanced glycation end products^6^, but there is still no unified theory. Because of the increasing incidence of PDR year by year, the prevention of the occurrence and development of PDR is the current research hotspot. However, studies on the risk factors for the occurrence and development of PDR are rare. Therefore, this study aimed to reveal the risk factors of PDR in patients with Type-2 diabetes mellitus (T2DM) and their correlations with CMT, so as to provide an objective basis for the early prevention and treatment of PDR.

## METHODS

This is a retrospective study. Subjects A total of 300 patients with confirmed T2DM receiving treatment in Aier Eye Hospital (Taiyuan) from February 2019 to February 2022 were included and divided into a PDR group (observation group) and non-PDR group (control group) according to whether they suffered from complications of PDR, with 150 patients in each group.

### Ethical Approval

The study was approved by the Institutional Ethics Committee of Aier Eye Hospital (Taiyuan) (No.: EYETYYY-20190130-01; date: January 30, 2019), and written informed consent was obtained from all participants.

T2DM was diagnosed referring to the diagnostic criteria for diabetes proposed by the American Diabetes Association (ADA) in 2020: fasting blood glucose (FBG) ≥ 7.0 mmol/L; two-hour glucose tolerance test (2hGTT) ≥ 11.1 mmol/L; glycosylated hemoglobin ≥ 6.5% and random blood glucose ≥ 11.1 mmol/L with typical diabetic symptoms.^7^ The diagnostic criteria for PDR referred to the 6-staging method for DR of the Ocular Fundus Disease Group, Ophthalmology Branch, Chinese Medical Association (CMA) in 2014^8^: retinal neovascularization or vitreous hemorrhage; fibrous proliferation, accompanied by preretinal hemorrhage or vitreous hemorrhage; tractional retinal detachment combined with fibrous hyperplasia.

### Inclusion criteria:

- Patients meeting the diagnostic criteria for T2DM or PDR.
- Patients aged ≥ 18 years.
- Patients with no cognitive impairment but an ability of normal communication and text reading.
- Patients who were informed of this study and signed the informed consent.

### Exclusion criteria:

- Other types of diabetes, such as Type-1 diabetes and gestational diabetes.
- T2DM is associated with other diseases, such as blood system diseases and acute complications of diabetes.
- Other retinal diseases, such as retinal vein occlusion, eye tumors and hypertensive retinopathy.
- Mental diseases.
- Pregnant or lactating women.
- Poor compliance, lack of cooperation with researchers, incomplete clinical data, and loss to follow-up.

### Observation indexes

The general information and laboratory indexes of the patients were recorded. -

### General information

Age, gender, nationality, marital status, body height, body weight, body mass index (BMI), blood pressure, course of diabetes and CMT.

### Laboratory indexes

Blood glucose-related tests included FBG, two hours postprandial blood glucose (2hPBG), hemoglobin (Hb) and glycosylated hemoglobin A1c (HbA1c). Renal function-related tests included blood urea nitrogen (BUN), creatinine (Cr), uric acid (UA) and urinary albumin. Blood lipid-related tests included total cholesterol (TC), triglyceride (TG), high-density lipoprotein (HDL) and low-density lipoprotein (LDL). The laboratory indexes mentioned above were all detected in the laboratory of our hospital. All enrolled patients completed the above tests and data collection immediately after admission.

### CMT measurement and fundus examination

CMT was measured using an OCT instrument in the scanning image of the macular area. All measurements were carried out by the same experienced technician. The systematic ocular examinations of all subjects were performed by the same ophthalmologist and the results were recorded. The condition of the anterior segment was checked in case of un-dilated pupils, while the fundus was checked in case of dilated pupils for the diagnosis of PDR or non-PDR. When both eyes were diagnosed as PDR, CMT on the severer side was recorded.

### Statistical Analysis

Analysis was conducted using SPSS 21.0. The measurement data were expressed as mean ± standard deviation (*χ̅*± *S*), and compared between the groups with the independent sample *t* test. The enumeration data were expressed as frequency (%), and compared between the groups by the χ^2^ test. Logistic regression analysis was carried out on the screened statistically significant factors of diabetic patients with PDR to predict the independent risk factors of PDR in T2DM, and the ROC curve was used to compare the evaluating efficiency of each factor. Correlation analysis was performed using Pearson correlation. *P*< 0.05 was considered statistically significant.

## RESULTS

No significant differences were found in gender, age, body height, body weight or BMI between the two groups (*P* > 0.05). In the observation group, the systolic blood pressure and diastolic blood pressure were higher, the course of diabetes was longer, and the CMT was thicker compared with those in the control group, with statistically significant differences (*P*< 0.05), as seen in Table-I.

**Table-I:** Comparison of general data between the two groups.

Item	Observation group(n=150)	Control group(n=150)	t/χ^2^	P
Age (years)	65.30±5.73	65.27±5.81	0.040	0.968
Gender (male/female)	80/70	78/72	0.053	0.817
Body height (m)	1.62±0.08	1.63±0.08	0.731	0.465
Body weight(Kg)	85.72±3.19	86.17±3.37	1.209	0.228
BMI(Kg/m^2^)	32.82±3.30	32.73±3.24	0.254	0.800
Systolic blood pressure (mmHg)	158.61±5.31	147.97±4.74	18.307	0.000
Diastolic blood pressure(mmHg)	109.26±5.81	107.77±6.44	2.100	0.037
Course of diabetes (years)	11.95±2.44	10.50±1.63	6.063	0.000
CMT(μm)	276.85±5.51	258.51±3.77	33.638	0.000

The FBG, 2hPBG, HbA1c, BUN, Cr, UA, urinary albumin and TC levels in the observation group were significantly higher than those in the control group (*P* < 0.05), but no statistically significant differences were found in Hb, TG, HDL or LDL level between the two groups (*P* > 0.05), as shown in Table-II.

**Table-II:** Comparison of laboratory indexes between the two groups.

Laboratory index	Observation group	Control group	t	P
FBG(mmol/L)	9.77±1.90	9.29±1.33	2.547	0.011
2hPBG(mmol/L)	13.67±2.01	13.26±1.45	2.012	0.045
Hb(g/L)	123.44±8.82	124.73±10.37	1.157	0.248
HbA1c(%)	9.65±1.69	8.86±2.51	3.217	0.001
BUN(mmol/L)	8.58±1.58	8.16±1.33	2.504	0.035
Cr(μmo/L)	113.46±7.22	111.95±5.30	2.073	0.039
UA(μmo/L)	338.84±31.17	330.22±30.81	2.409	0.017
Urinary albumin(g/L)	165.81±11.11	161.07±10.45	3.813	0.000
TC(mmol/L)	4.86±1.77	4.50±1.24	2.031	0.043
TG(mmol/L)	1.46±0.38	1.52±0.32	1.417	0.157
HDL(mmol/L)	1.49±0.48	1.43±0.49	1.064	0.288
LDL(mmol/L)	2.49±0.65	2.52±0.62	0.399	0.690

The binary logistic regression analysis was conducted with PDR as the independent variable, and systolic blood pressure, diastolic blood pressure, course of diabetes, FBG, 2hPBG, HbA1c, BUN, Cr, UA, urinary albumin and TC as covariants. The results showed that systolic blood pressure, diastolic blood pressure, course of diabetes, FBG, 2hPBG and urinary albumin were independent risk factors for T2DM complicated with PDR (Table-III).

**Table-III:** Logistic regression analysis of risk factors for PDR in T2DM patients.

Index	β	SE	Waldχ^2^	P	OR	95.0%CI
Systolic blood pressure	-0.510	0.063	65.869	0.000	0.600	0.531~0.679
Diastolic blood pressure	0.128	0.044	8.222	0.004	1.136	1.041~1.240
Course of diabetes	-0.275	0.131	4.393	0.036	0.760	0.587~0.982
FBG	-3.173	0.783	16.403	0.000	0.042	0.009~0.194
2hPBG	2.764	0.714	14.975	0.000	15.855	3.911~64.276
HbA1c	-0.078	0.097	0.659	0.417	0.925	0.765~1.117
BUN	-0.204	0.162	1.596	0.206	0.815	0.594~1.119
Cr	-0.029	0.038	0.571	0.450	0.971	0.901~1.047
UA	-0.017	0.009	3.353	0.067	0.983	0.966~1.001
Urinary albumin	-0.058	0.024	6.119	0.013	0.943	0.901~0.988
TC	-0.112	0.148	0.571	0.450	0.894	0.669~0.988

**Fig.1:**
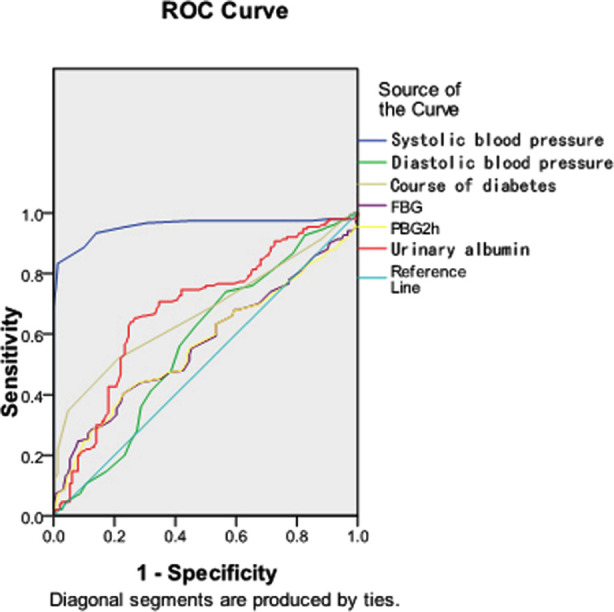
Prediction model and single-factor ROC curve.

Using the independent risk factors obtained in logistic regression analysis, the ROC curve of the correlation factor model combined with PDR was established. ROC curve revealed that systolic blood pressure, course of diabetes and urinary albumin could better predict the occurrence of PDR, as presented in Table-IV and Fig.1.

**Table-IV:** Diagnostic significance of independent risk factors in T2DM patients with PDR.

Index	Sensitivity	Specificity	AUC	Youden’s index	95.0%CI	P
Systolic blood pressure	0.833	0.987	0.955	0.820	0.929~0.982	0.000
Diastolic blood pressure	0.740	0.433	0.571	0.173	0.505~0.636	0.035
Course of diabetes	0.520	0.787	0.673	0.307	0.612~0.734	0.000
FBG	0.247	0.920	0.570	0.167	0.505~0.635	0.037
2hPBG	0.400	0.773	0.566	0.173	0.500~0.631	0.050
Urinary albumin	0.640	0.747	0.682	0.387	0.620~0.743	0.000

**Table-V:** Correlation analysis of independent risk factors with CMT.

	Systolic blood pressure(mmHg)		Course of diabetes(years)		HbA1c(%)		Urinary albumin(g/L)	

	r	P	r	P	r	P	r	P
CMT	0.642	0.000	0.190	0.0001	0.210	0.000	0.238	0.000

Correlation analysis demonstrated that CMT was positively correlated with systolic blood pressure, course of diabetes, HbA1c level and urinary albumin level, indicating that the increase in systolic blood pressure, course of diabetes, HbA1c level and urinary albumin level will significantly increase CMT in the patients.

## DISCUSSION

This study found that the increase in blood pressure was correlated with the occurrence of PDR in patients with T2DM. We believe that it may be caused by the automatic pressure regulation of retinal microvessels is damaged due to hyperglycemia, which makes hypertension more likely to damage vascular endothelial cells. When blood pressure, especially systolic blood pressure, rises, the damage to vascular endothelial cells will continue to exist, thereby leading to increased platelet attachment, and resulting in no blood flow in retinal vessels, Finally, neovascularization occurs due to tissue hypoxia. The British diabetes expert consensus^9^ points out that the risk of retinopathy increases in patients with diabetes combined with hypertension, which is the same as our results.

Previous studies^10^,^11^ have shown that the occurrence and severity of PDR are related to the course of diabetes, and with the increase in the course of diabetes, the probability of PDR in patients increases significantly. Our results revealed that the course of diabetes was an important risk factor for PDR, which is in line with previous research results. The main factor affecting the progression of PDR is the control of the blood glucose level of the patients. Good blood glucose control is the basis for reducing the occurrence and development of PDR. In the clinic, FBG and 2hPBG levels are usually used to evaluate the blood glucose control of patients, and HbAlc to evaluate their average blood glucose value over a period of time and determine the quality of blood glucose control.

A relevant study^12^ has demonstrated that hyperglycemia can cause vascular endothelial damage, which damages the blood-retinal barrier and narrows the retinal microvascular lumina, thus ultimately affecting the retinal blood flow, and promoting the occurrence and progression of PDR. The results of this study also suggested that FBG and 2hPBG levels were risk factors for PDR in patients with T2DM, which is consistent with relevant reports.^13^

Many studies at home and abroad^14^-^16^ have found that PDR is closely related to the rapid decrease of glomerular filtration rate. Diabetic patients with PDR are more prone to proteinuria than diabetic patients without PDR, and the level of proteinuria is positively correlated with the severity of DR. It has also been confirmed^17^ that PDR is a risk factor for diabetic nephropathy (DN), which in turn has a great predictive effect on PDR. Our results showed that the level of urinary albumin was an independent risk factor for the occurrence of PDR, which is in line with previous reports.^18^ The specific mechanism is speculated to be that the occurrence of PDR has the same pathological basis as DN, for example, abnormal proteins produce a large number of free radicals, which leads to the apoptosis of retinal capillary endothelial cells.

The choroidal capillary layer provides nutrients and metabolic exchange for the outer layer of the retina, while the macular fovea lacks the supply of the retinal artery. If the choroidal capillaries in the macular fovea are damaged, it will affect the macular function, affecting the visual function of the patients. Moreover, hyperglycemia and hypertension can cause damage to choroidal capillaries, resulting in foveal edema and ultimately changes in CMT.^19^,^20^ The present study showed that CMT was positively correlated with systolic blood pressure, course of diabetes, HbA1c level and urinary albumin level indicating that CMT increases with the increase in systolic blood pressure, course of diabetes, HbA1c level and urinary albumin level.

It is currently a hot spot in clinical research to predict the risk factors of diseases by establishing relevant models based on big data. The establishment of disease-related models can predict the risk factors for its occurrence and play an important role in disease prevention. This study suggests that for patients with T2DM. One should pay attention to blood pressure, blood lipid, and hepatic and renal functions should be paid attention to, and the occurrence and development of PDR in clinical practice should be specifically monitored and prevented while testing blood glucose-related indexes.

### Limitation

It includes small sample size, the risk factors are not stratified, and some results are biased. Therefore, the analysis in this study still needs to be further confirmed.

## CONCLUSIONS

The important risk factors of PDR in patients with T2DM include hypertension, course of diabetes, FBG, 2hPBG and renal function-related indexes, suggesting that the risk of retinal involvement increases in patients with hypertension, hyperglycemia and DN. Therefore, attention should be paid to the control of blood pressure, hyperglycemia and DN.

### Authors’ Contributions:

**HH:** Carried out the studies, participated in collecting data, drafted the manuscript, are responsible and accountable for the accuracy and integrity of the work.

**SJ:** Performed the statistical analysis and participated in its design.

**HN:** Participated in acquisition, analysis, interpretation of data and drafting the manuscript.

All authors read and approved the final manuscript.
